# Sir Benjamin Brodie

**Published:** 1861-03

**Authors:** 


SIR BENJAMIN BRODIE underwent an operation for the extraction of cataract, on the twenty-first of January. The progress of the case was favorable, up to the latest accounts.

